# Discovery of novel neutral glycosphingolipids in cereal crops: rapid profiling using reversed-phased HPLC–ESI–QqTOF with parallel reaction monitoring

**DOI:** 10.1038/s41598-023-49981-7

**Published:** 2023-12-19

**Authors:** Dingyi Yu, Berin A. Boughton, Thusitha W. T. Rupasinghe, Camilla B. Hill, Cornelia Herrfurth, Patricia Scholz, Ivo Feussner, Ute Roessner

**Affiliations:** 1 School of BioSciences, University of Melbourne, Parkville, VIC 3010 Australia; 2 Mass Spectrometry Facility, St Vincent Institute of Medical Research, Fitzroy, VIC 3065 Australia; 3 Australian National Phenome Centre, Murdoch University, Murdoch, WA 6157 Australia; 4 Department of Animal, Plant and Soil Sciences, La Trobe Institute for Sustainable Agriculture and Food, La Trobe University, Bundoora, VIC 3083 Australia; 5 AbSciex, 2 Gilda Court, Mulgrave, VIC 3170 Australia; 6 Western Barley Genetics Alliance, Western Australian State Agricultural Biotechnology Centre, School of Veterinary and Life Sciences, Murdoch University, Murdoch, WA 6157 Australia; 7 Department of Plant Biochemistry, Albrecht-Von-Haller-Institute for Plant Sciences, University of Goettingen, Justus-Von-Liebig Weg 11, 37077 Goettingen, Germany; 8 ENS Lyon-Laboratoire Reproduction et Développement des Plantes, Equipe Signalisation Cellulaire (SICE), 46, Allée d’Italie, 69364 Lyon Cedex 07, France; 9 Service Unit for Metabolomics and Lipidomics, Goettingen Center for Molecular Biosciences (GZMB), University of Goettingen, Justus-Von-Liebig Weg 11, 37077 Goettingen, Germany; 10 Department of Plant Biochemistry, Goettingen Center for Molecular Biosciences (GZMB), University of Goettingen, Justus-Von-Liebig Weg 11, 37077 Goettingen, Germany; 11 Research School of Biology, Australian National University, Acton, ACT 2601 Australia

**Keywords:** Lipidomics, Plant stress responses

## Abstract

This study explores the sphingolipid class of oligohexosylceramides (OHCs), a rarely studied group, in barley (*Hordeum vulgare* L.) through a new lipidomics approach. Profiling identified 45 OHCs in barley (*Hordeum vulgare* L.), elucidating their fatty acid (FA), long-chain base (LCB) and sugar residue compositions; and was accomplished by monophasic extraction followed by reverse-phased high performance liquid chromatography electrospray ionisation quadrupole-time-of-flight tandem mass spectrometry (HPLC–ESI–QqTOF–MS/MS) employing parallel reaction monitoring (PRM). Results revealed unknown ceramide species and highlighted distinctive FA and LCB compositions when compared to other sphingolipid classes. Structurally, the OHCs featured predominantly trihydroxy LCBs associated with hydroxylated FAs and oligohexosyl residues consisting of two–five glucose units in a linear 1 → 4 linkage. A survey found OHCs in tissues of major cereal crops while noting their absence in conventional dicot model plants. This study found salinity stress had only minor effects on the OHC profile in barley roots, leaving questions about their precise functions in plant biology unanswered.

## Introduction

**Figure 1:**
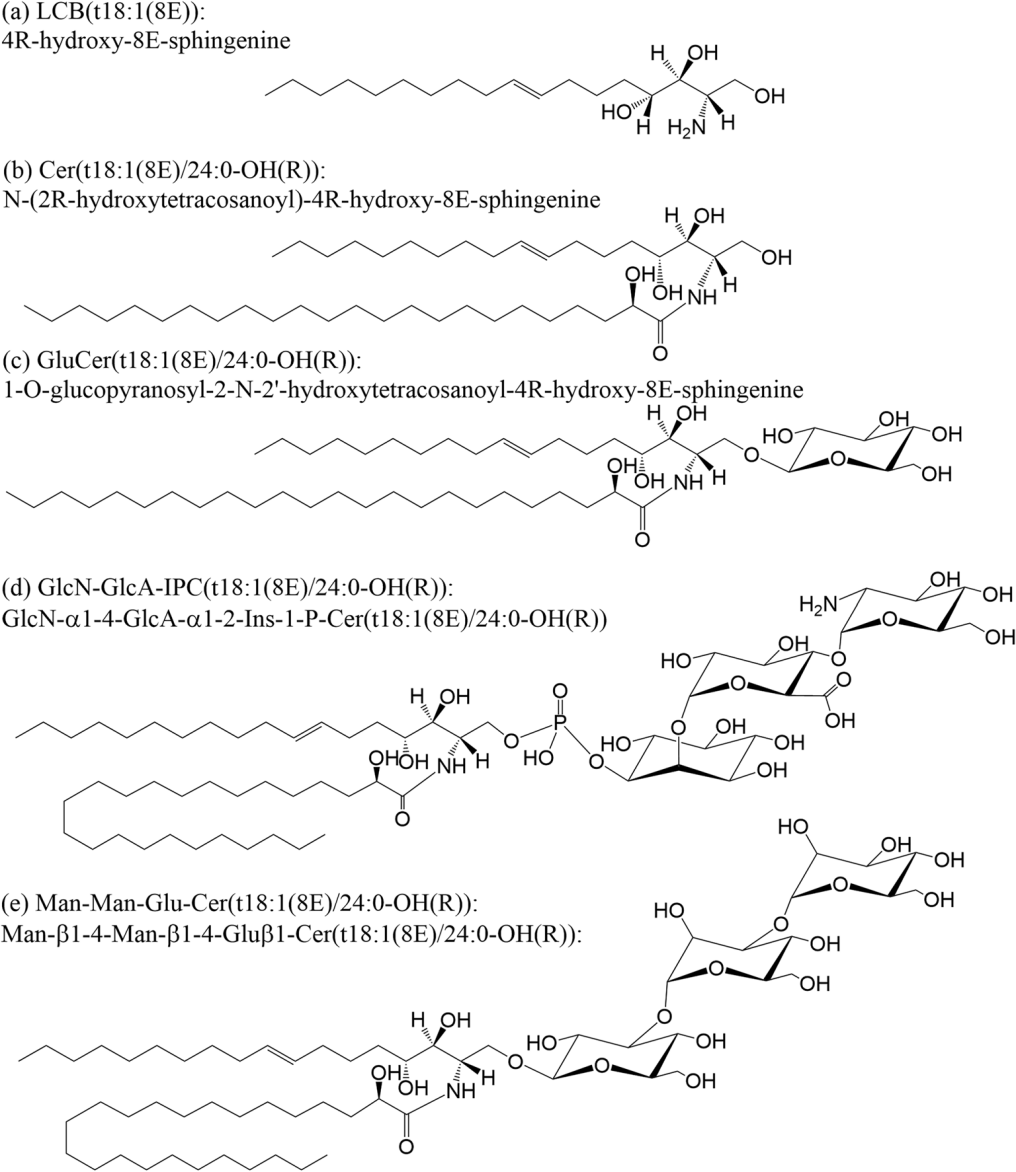
Molecular structures of five representative plant sphingolipid classes (Nomenclature from LIPID MAPS^45^). (**a**) A free long chain base, LCB(t18:1(8E)); (**b**) A ceramide (Cer), Cer(t18:1(8E)/24:0-OH(R)); (**c**) A monoglycosylceramide (MonoGlcCer), GluCer(t18:1(8E)/24:0-OH(R)); (**d**) A GIPC, GluN-GluA-IPC(t18:1(8E)/24:0-OH(R)). (e) A trihexosylceramide (TriHexCer), Man-Man-Glu-Cer(t18:1(8E)/24:0-OH(R)).

Sphingolipids constitute a broad, multifaceted lipid class integral to plant membrane systems. They have pivotal roles in various fundamental processes, encompassing membrane structural maintenance, programmed cell death regulation, and the orchestration of abscisic acid (ABA)-triggered signalling pathways in response to diverse environmental stresses^1–4^. Plant sphingolipids can be divided into four major classes differing in their structural complexity: free long-chain bases (LCB), ceramides (Cer), glycosylceramides (GlcCer) and glycosyl inositolphosphoceramides (GIPC) (Fig. 1)^1–3^. Plant GlcCers are comprised of one or multiple glycosyl residues as a polar headgroup attached to the C1 position of a ceramide backbone. One type of monoglycosylceramide—glucosylceramides (GluCers) carrying a β-d-glucosyl headgroup have been found widely distributed in many plants^4–6^ (Fig. 1). Monoglucosylceramides are essential components of the outer monolayer of the plasma membrane (PM), accounting for 5–30 mol % of total lipid in the PM^7,8^.

Further additions of glycosyl residues lead to the formation oligohexosylceramides (OHCs) including di-, tri-, tetra- and pentahexosylceramides (DiHexCers, TriHexCers, TetraHexCers and PentaHexCers). Previously, OHCs were detected and characterised in only a few cereal tissues including wheat^9^ and rice grains^10^. In wheat, the GluCer were found to be mannosylated, forming a [Man(βl → 4)]_1–3_-Glu(β1 → 4)-Cer structure (Fig. 1e). In rice, a more complex range of molecular structures was observed, Glu(β1 → 4)-Glu(β1 → 4)-Cer, Glu(β1 → 4)-Man(βl → 4)-Glu(β1 → 4)-Cer and Glu(β1 → 4)-[Man(βl → 4)]_2_-Glu(β1 → 4)-Cer in addition to those in wheat^10^. The total concentration of Di-, Tri- and TetraHexCers was observed to be less than 10% of the total concentration of monohexosylceramides (HexCer) in wheat grain extract^9^. Apart from cereals, only small amounts of DiHexCer have been reported in spinach leaves^11^. Despite their discovery in the 1980s, there is a notable absence of subsequent studies examining any OHC species *in planta*, and our understanding of their functions within plants remains limited.

To expand our understanding, our objective was to specifically identify OHCs in barley (*Hordeum vulgare* L.) and uncover their structural characteristics. This involved the establishment of a modern reverse-phased (RP) high performance liquid chromatography electrospray ionisation quadrupole-time-of-flight tandem mass spectrometry (HPLC–ESI–QqTOF) methodology using parallel reaction monitoring (PRM)^12^ (see Supplementary Fig. S1).

OHC profiling has previously required laborious procedures including isolation from bulk plant material (> 1 kg fresh tissue) followed by degradation to individual components, then individual analyses of specific fatty acid (FA), LCB and glycosidic components^9,11,13^. In addition, identification of individual OHC species with specific LCB and FA associations was difficult to achieve due to the separate analysis requirements for LCBs and FAs^9,10,14^. Modern mass spectrometers are capable of advanced metabolite identification and structural elucidation via tandem-mass spectrometry (MS/MS) methods. In this study, we developed a rapid, stable profiling MS/MS method that can be performed in just a few hours, using small quantities of plant tissue (~ 200 mg) and a monophasic lipid extraction. To account for potential variations in the sugar residue composition, we incorporated monosaccharide glycan analysis through acid hydrolysis coupled to gas chromatography mass spectrometry (GC–MS) profiling.

Lipid composition may be linked to a plant’s ability to adapt to and withstand salt stress^15–17^. OHCs, which are present in barley roots but absent in the salt-sensitive model plant *Arabidopsis* may be associated with their contrasting ability to tolerate salinity. To examine roles of OHCs in response to salinity stress, we investigated OHC compositional changes in barley roots under salt stress. Comprehensive profiling of OHCs across a diverse range of plant species revealed the presence of OHCs in the agriculturally most important cereal crops, wheat, rice, and barley, along with other grasses. Notably, OHCs were absent in other plant families, especially dicots, included in our testing.

## Results

### Detection and characterisation of OHCs in barley roots

**Figure 2:**
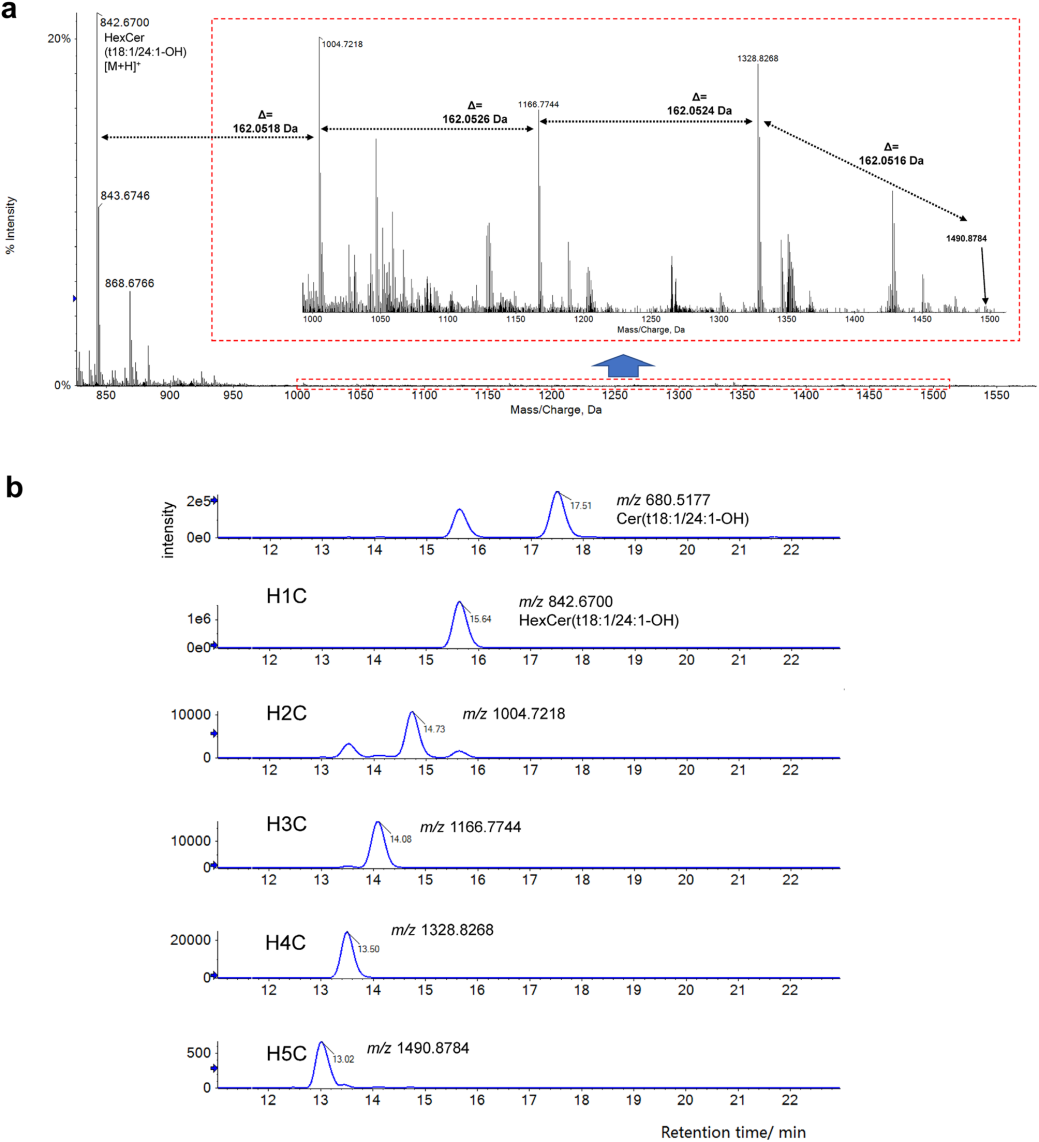
MS1 spectra and extracted ion chromatograms (EIC) of oligohexosylceramides (OHCs). (**a**) Average MS1 spectra obtained from HPLC–ESI–QqTOF exhibited a sequence of cations with mass difference close to a dehydrated hexose (C_6_H_10_O_5_, *m/z* 162.0528). (**b**) EIC displayed elution times which decreased with increasing *m/z* value among these ions.

In previous studies, examining barley root lipids, we detected a series of unidentified ions within the mass-to-charge ratio (*m/z*) range 800–1500, eluting between 10–16 min (Fig. 2a)^12^. Remarkably, the average mass spectra showed a sequence of cations, *m/z* 842.6700, 1004.7218, 1166.7744, 1328.8268 and 1490.8784, with average mass defects between ions (162.0516–162.0524 Da), matching to the loss of a dehydrated hexose sugar residue (i.e. C_6_H_10_O_5_, Δ 162.0528 Da) with a mass error below 7.5 ppm. Extracted ion chromatograms (EIC) of the various ions demonstrated decreasing elution times with increasing *m/z* (Fig. 2b), according with the chromatographic behaviour of molecules containing multiple sugar units when separated using RP C18 chromatography^18^. Increasing sugar units in a molecule render the molecule more hydrophilic, leading to a weaker interaction with the RP and a subsequent earlier elution.

Previously^12^, the ion *m/z* 842.6700 at 15.64 min was confirmed as protonated HexCer(t18:1/24:1-OH) with a mass error of -2.8 ppm. Thus, unknown compounds with *m/z* 1004.7218 (compound named as ‘H_2_C’), 1166.7744 (H_3_C), 1328.8268 (H_4_C) and 1490.8784 (H_5_C) were predicted to be protonated precursors carrying an additional 2–5 hexose units with the same ceramide backbone at the reducing end with a mass error of − 3.2, − 3.1, − 3.2 and − 3.1 ppm respectively.

**Figure 3:**
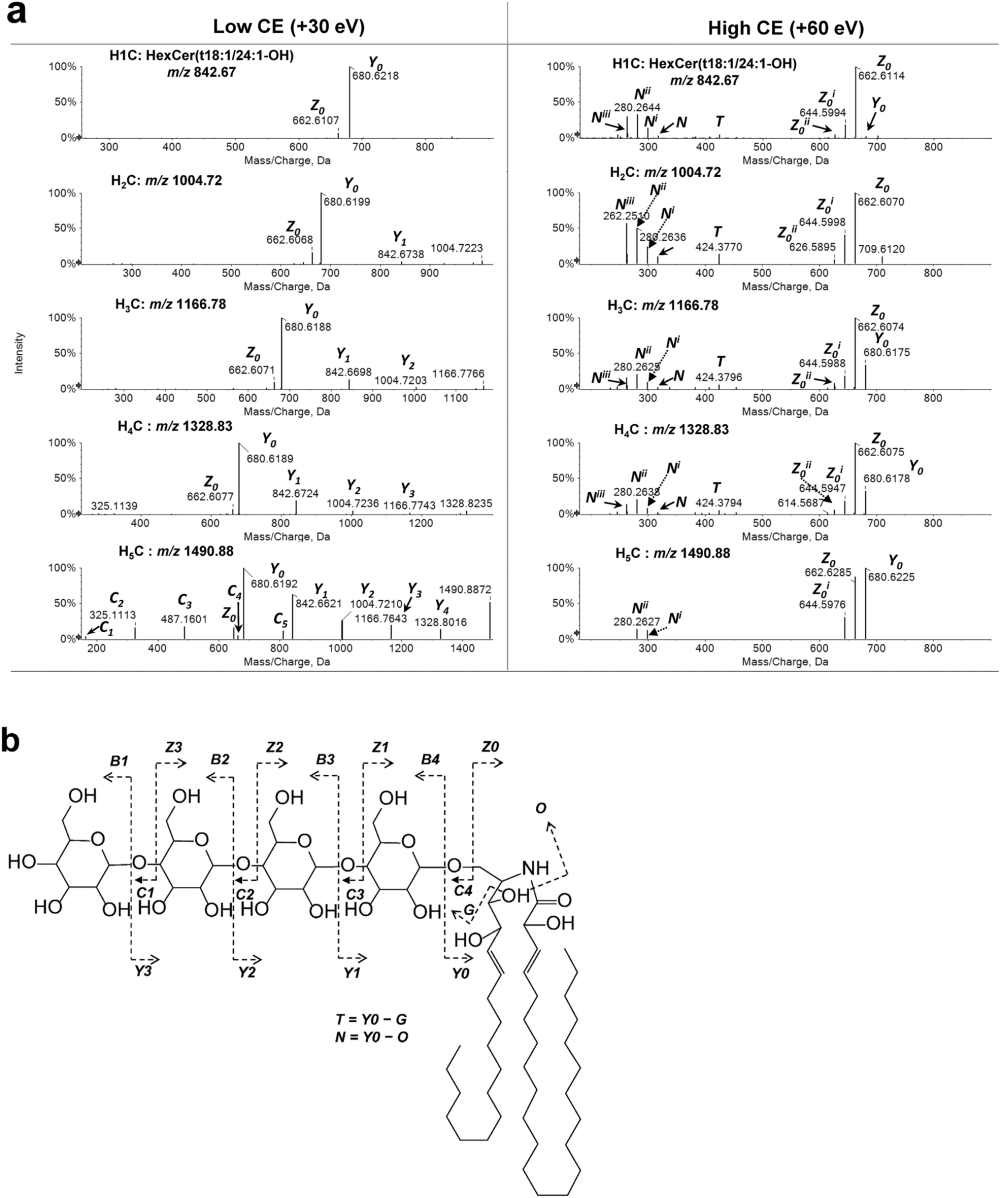
Positive-ion MS/MS spectra of predicted OHCs and proposed cleavages under collision-induced dissociation. (**a**) MS/MS spectra of HexCer(t18:1/24:1-OH) and predicted Di-, Tri-, Tetra- and PentaHexCer (H_2~5_C) in ESI^+^ under low (+ 30 eV) and high (+ 60 eV) collision energies. (**b**) Scheme showing proposed fragmentations of the precursor TetraHexCer(t18:1/24:1-OH). Positions of double bonds and hydroxyl groups in ceramide residues are hypothetical.

To verify the predicted molecular structures of H_2-5_C, we carried out MS/MS experiments using collision induced dissociation (CID) to collect MS/MS spectra in both ESI^+^ and ESI^−^ modes. In ESI^+^ mode, low (+ 30 eV) and high (+ 60 eV) CEs were employed to dissociate protonated precursors (Fig. 3).

MS/MS spectra of H_2–5_Cs under low CE showed prominent cations at *m/z* 680.619 and 662.608, matching those of H_1_C. Both *Y*_*0*_ and *Z*_*0*_ ions were observed in MS/MS spectra of H_1_C corresponding to the protonated Cer(t18:1/24:1-OH) (C_42_H_82_NO_5_, *m/*z 680.6193) and its associated dehydrate (C_42_H_80_NO_4_, *m/*z 662.6087) mass error below 1 ppm, resulting from cleavage of glycosidic bonds between the ceramide and headgroup (Fig. 3b). Larger fragments in MS/MS spectra of H_2-5_Cs with one or several Δ ~ 162.0500 Da differences from *Y*_*0*_ were predicted to be other *Y* or *Z* type ions from the cleavage of internal glycosidic bonds between hexose units. For example, in MS/MS spectra of H_5_C (Fig. 3), *m/z* 1328.8216, 1166.7743, 1004.7210, 842.6711 were predicted to be *Y* series ions arising from cleavage of 1–5 hexose units on the non-reducing end with a mass error of − 7.7, − 3.0, − 1.5, − 1.2 ppm respectively. Compared to *Y* ions, *Z* series ions were observed in the MS/MS spectra of H_2-5_C with low abundance. Apart from glycosylated ceramide cations (*Y* and *Z* ions), alkali-metal/protonated saccharide ions (*B* and *C* ions) were also observed in MS/MS spectra of H_2-5_Cs. Information from *C* ions can reveal the number of hexoses in the oligosaccharide residue. For example, *C* series ions in the MS/MS spectra of H_5_C were 163.0603, 325.1113, 487.1601, 649.2180 and 811.2677, corresponding to hexose (C_6_H_11_O_5_, *C*_*1*_), dihexose (C_12_H_21_O_10_, *C*_*2*_), trihexose (C_18_H_31_O_15_, *C*_*3*_), tetrahexose (C_24_H_41_O_20_, *C*_*4*_) and pentahexose (C_30_H_51_O_25_, *C*_*5*_) with a mass error below 10 ppm.

The presence of *Y*_*0-4*_, *Z*_*0-4*_ and *C*_*1-5*_ ions in H_5_C also indicate the linear structure of sugar residues, as at least one ion in each above series would be missing in the MS/MS spectrum of a branched oligosaccharide^19^. MS/MS spectra of H_1_C at high CE + 60 eV contain rich clusters of fragments, *N* and *Y*_*0*_*/Z*_*0*_ series: *m/z* 316.2845 (*N*), 298.2753 (*N*^*i*^), 280.2644 (*N*^*ii*^), 262.2542 (*N*^*iii*^) and *m/z* 680.6185 (*Y*_*0*_), 662.6114 (*Z*_*0*_), 644.5994 (*Z*_*0*_^*i*^) and 626.5878 (*Z*_*0*_^*ii*^) were protonated LCB and ceramide backbone with corresponding dehydrates from up to three dehydration processes with a mass error below 7.1 ppm. The number of *N* dehydrates reflects the number of hydroxyls found on the LCB. Under appropriate conditions, HexCer species containing trihydroxy LCBs can have up to three *N* dehydrates from losses of 1–3 hydroxy groups; while a dihydroxy LCB could only result in up to two *N* dehydrates. At CE + 60 eV, all of the above *N* and *Y*_*0*_*/Z*_*0*_ series ions from H_1_C appeared in the MS/MS spectra of H_2-5_C (Fig. 3a). This further verified that H_2-5_C were likely composed of the same ceramide conjugated with different oligosaccharides. Apart from *Y*_*0*_ and *N*/*Z* series of ions, a small amount of *m/z* 424.379 (*T* ion) was observed in the MS/MS spectrum of H_1-5_C, which was predicted to be from the cleavage in LCB (C_26_H_50_NO_3_, *m/z* 424.3791, mass error below 1 ppm).

In negative-ion mode, MS/MS spectra of deprotonated H_2-5_C, fragments from the glycosidic bond cleavage (*Y*/*Z* and *B*/*C* ions) and cross-ring cleavage (*A*/*X* ions) were abundant since the negative charge tends to be retained on sugar residues after dissociation. These ions confirmed the sequence and branching type derived from positive-ion MS/MS spectra and further provided partial linkage information.

**Figure 4:**
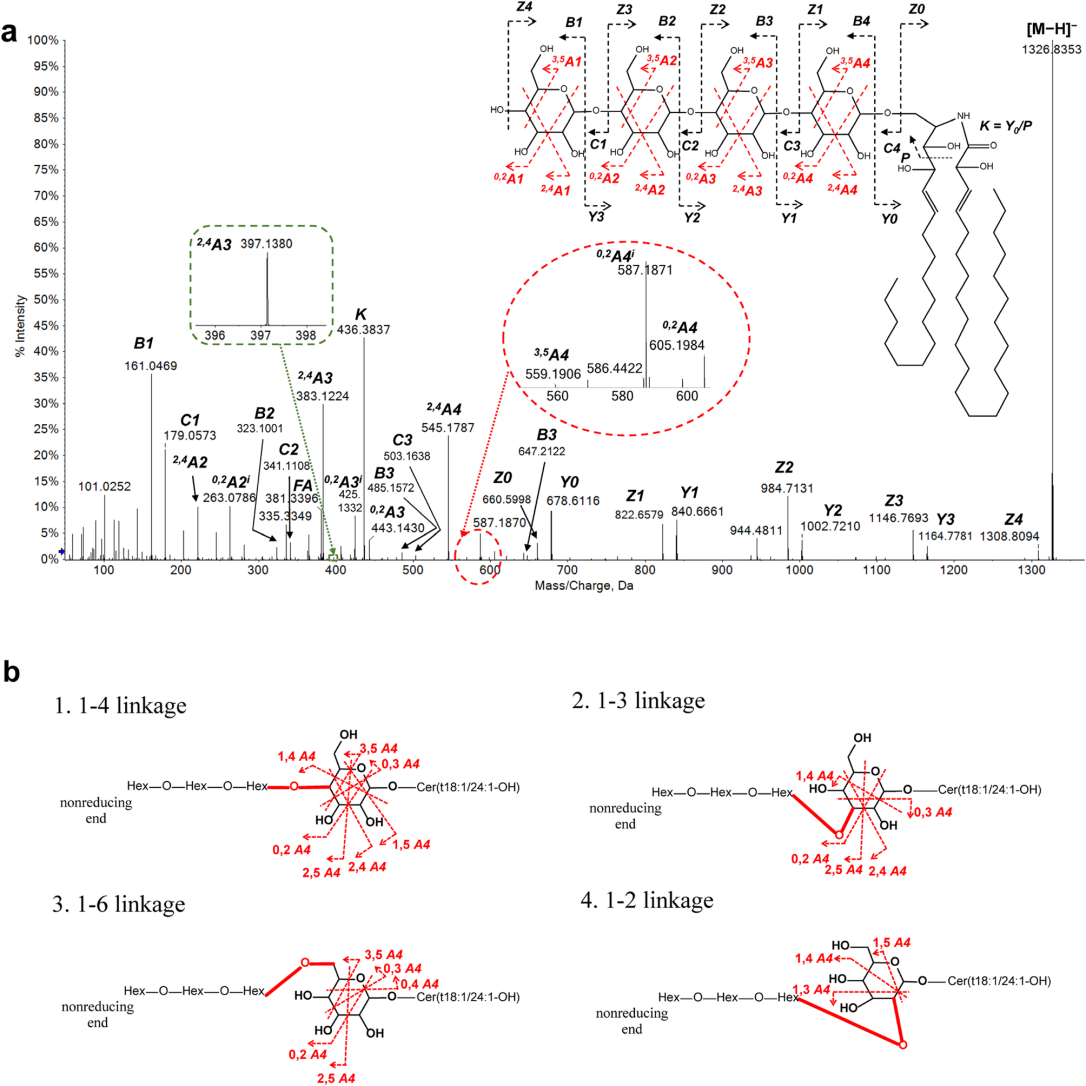
(**a**) MS/MS spectra of TetraHexCer(t18:1/24:1-OH) in ESI^−^ mode and fragment nomenclature. (**b**) Theoritical cross-ring fragmentation pattern of the fourth hexose unit from the non-reducing end for each different linkage position (1 → 4, 1 → 3, 1 → 6, 1 → 2).

First, the presence of linear oligosaccharide in H_2-5_C were further verified by *Y*/*Z* and *B*/*C* ions in negative-mode MS/MS spectra (Fig. 4a). For example, abundant *Y* ions (*m/z* 1164.7805 (*Y*_*3*_), 1002.7264 (*Y*_*2*_), 840.6703 (*Y*_*1*_) and 678.6132 (*Y*_*2*_)), *Z* ions (*m/z* 1146.7684 (*Z*_*3*_), 984.7142 (*Z*_*2*_), 822.6579 (*Z*_*1*_), 660.6063 (*Z*_*0*_)), *B* ions (*m/z* 161.0468 (*B*_*1*_), 323.0996 (*B*_*2*_), 485.1524 (*B*_*3*_), 647.2052(*B*_*4*_)) and *C* ions (*m/z* 179.0573 (*C*_*1*_), 341.1101 (*C*_*2*_), 503.1629 (*C*_*3*_), 665.2157 (*C*_*4*_)) were observed with a mass error below 6.5 ppm in MS/MS spectra of H_4_C at a CE –70 eV.

Second, apart from sugar residues, an abundant fragment *K* ion at *m/z* 436.3837 was observed in negative-ion MS/MS spectra of H_1-5_C under a CE − 80 ± 20 eV (Fig. 4a). It is predicted from the cleavage of *Y*_*0*_ between C3 and C4 position of the t18:1 LCB chain followed by a dehydration process with a mass error of 5.1 ppm.

Third, glycosidic linkage information on oligosaccharide residues can also be partially diagnosed from various *A*-type cross-ring fragments. *A*-type fragments arise from a single cleavage on the sugar ring with charges retained on the non-reducing terminus of the sugar^19^.

The existence of diagnostic ^*3,5*^*A* ions (i.e., ^*3,5*^*A4*, *m/z* 559.191, mass error 5.4 ppm) in the negative-ion MS/MS spectra ruled out the possibility of 1 → 3 and 1 → 2 linkage position between hexose units (Table 1, Fig. 4b). Since 1 → 6 linkage was previously found to form branching topology within oligosaccharide structures^20^ and the structure of oligosaccharide residues in barley OHCs is linear as described above^19^, the linkage position between hexoses is more likely to be 1 → 4.

**Table 1:** Potential diagnostic cross-ring *A4* ions in ESI^−^ mode for each potential linkage position (1 → 4, 1 → 3, 1 → 6, 1 → 2) between the third and fourth hexose unit from the non-reducing end of TetraHexCer(t18:1/24:1-OH).

	1 → 4	1 → 3	1 → 6	1 → 2
^0,2^*A4*	605.16	605.19	605.19	N.A
^0,3^*A4*	575.18	N.A	575.18	N.A
^0,4^*A4*	N.A	N.A	545.17	N.A
^1,3^*A4*	N.A	545.17	N.A	545.17
^1,4^*A4*	575.18	575.18	N.A	575.18
^2,4^*A4*	545.17	545.17	N.A	N.A
^2,5^*A4*	589.20	589.20	589.20	N.A
^3,5^*A4*	559.19*	N.A	559.19*	N.A

*Existence of ^3,5^*A4* with a *m/z* of 559.19 rules out the possibility of 1 → 2 and 1 → 3 linkages.

*N.A.* not applicable.

In summary, we have identified and characterized OHCs in barley tissue, with some OHCs containing up to five linear hexosyl residues. To the best of our knowledge, this marks the first report of OHCs bearing five glycosyl units in any plant species.

### Screening and profiling of OHCs in barley roots using PRM assays

The length of fatty acyl chains, degree of unsaturation and hydroxylation of fatty acids usually lead to a large diversity of the ceramide backbones found in sphingolipids. To screen other potential Di, Tri-, Tetra- and PentaHexCers containing different ceramide backbones, we employed a newly developed parallel reaction monitoring (PRM) assay combining high-resolution MS1 scan and high-resolution MS/MS experiments on HPLC–ESI–QqTOF. OHCs were separated by RP then analysed using HRMS. Chromatographic conditions and ESI source settings were further optimised from the previous methodology used to detect and characterise OHCs^12^. Different combinations of LCB, FA and oligohexosyl residue in OHCs can be resolved by chromatographic retention time and/or high-resolution MS/MS. Methodologically, a targeted list for PRM assays containing theoretical Di-, Tri-, Tetra-, PentaHexCers was compiled for screening OHCs in barley roots. 188 entries of precursors were created by adding two to five hexose units to 47 ceramide backbones previously detected in Cers and HexCers^12^. Multiple PRM screening assays employing a subset of the 188 predicted ions was used to confirm the presence of 45 OHCs, including 12 DiHexCer, 12 TriHexCer, 12 TetraHexCer and 9 PentaHexCer species, in barley roots using this approach (Table 2, Supplementary Table S2). The established PRM method allowed profiling of the 45 OHCs and their corresponding HexCers in barley root extract based on high-resolution MS1 data. The extracted ion chromatograms (EIC) of precursors were used for semi-quantification after integration. Precursor types used for profiling were [M + H]^+^ for Mono-, Di- and TriHexCers and [M + NH_4_ + H]^2+^ for Tetra- and PentaHexCers.

**Table 2:** Formula, precursor ions and retention times (RTs) of oligohexosylceramides (OHCs) profiled in barley roots using HPLC–ESI–QqTOF.

No	Compound	Formula	[M + H]^+^	[M + NH_4_ + H]^2+^	RT
1	DiHexCer(t18:1/26:1-OH)	C_56_H_105_NO_15_	1032.7562	525.3953	16.4
2	TriHexCer(t18:1/26:1-OH)	C_62_H_115_NO_20_	1194.8090	606.4217	15.5
3	TetraHexCer(t18:1/26:1-OH)	C_68_H_125_NO_25_	1356.8618	687.4481	14.9
4	PentaHexCer(t18:1/26:1-OH)	C_74_H_135_NO_30_	1518.9146	768.4745	14.3
5	DiHexCer(t18:1/24:1-OH)	C_54_H_101_NO_15_	1004.7250	511.3797	14.7
6	TriHexCer(t18:1/24:1-OH)	C_60_H_111_NO_20_	1166.7780	592.4062	14.1
7	TetraHexCer(t18:1/24:1-OH)	C_66_H_121_NO_25_	1328.8310	673.4327	13.5
8	PentaHexCer(t18:1/24:1-OH)	C_72_H_131_NO_30_	1490.8830	754.4587	13.0
9	DiHexCer(t18:1/22:1-OH)	C_52_H_97_NO_15_	976.6936	497.3640	13.3
10	TriHexCer(t18:1/22:1-OH)	C_58_H_107_NO_20_	1138.7464	578.3904	12.7
11	TetraHexCer(t18:1/22:1-OH)	C_64_H_117_NO_25_	1300.7992	659.4168	12.1
12	PentaHexCer(t18:1/22:1-OH)	C_70_H_127_NO_30_	1462.8520	740.4432	11.5
13	DiHexCer(t18:1/20:1-OH)	C_50_H_93_NO_15_	948.6623	483.3483	11.8
14	TriHexCer(t18:1/20:1-OH)	C_56_H_103_NO_20_	1110.7151	564.3747	11.1
15	TetraHexCer(t18:1/20:1-OH)	C_62_H_113_NO_25_	1272.7679	645.4011	10.5
16	PentaHexCer(t18:1/20:1-OH)	C_68_H_123_NO_30_	1434.8207	726.4275	9.9
17	DiHexCer(t18:1/26:0-OH)	C_56_H_107_NO_15_	1034.7719	526.4031	18.6
18	TriHexCer(t18:1/26:0-OH)	C_62_H_117_NO_20_	1196.8247	607.4295	17.3
19	TetraHexCer(t18:1/26:0-OH)	C_68_H_127_NO_25_	1358.8775	688.4559	16.5
20	PentaHexCer(t18:1/26:0-OH)	C_74_H_137_NO_30_	1520.9303	769.4823	15.8
21	DiHexCer(t18:1/24:0-OH)	C_54_H_103_NO_15_	1006.7407	512.3875	16.3
22	TriHexCer(t18:1/24:0-OH)	C_60_H_113_NO_20_	1168.7937	593.4140	15.4
23	TetraHexCer(t18:1/24:0-OH)	C_66_H_123_NO_25_	1330.8467	674.4405	14.8
24	PentaHexCer(t18:1/24:0-OH)	C_72_H_133_NO_30_	1492.8987	755.4665	14.3
25	DiHexCer(t18:1/22:0-OH)	C_52_H_99_NO_15_	978.7094	498.3719	14.6
26	TriHexCer(t18:1/22:0-OH)	C_58_H_109_NO_20_	1140.7624	579.3984	14.0
27	TetraHexCer(t18:1/22:0-OH)	C_64_H_119_NO_25_	1302.8154	660.4249	13.4
28	PentaHexCer(t18:1/22:0-OH)	C_70_H_129_NO_30_	1464.8674	741.4509	12.8
29	DiHexCer(t18:1/20:0-OH)	C_50_H_95_NO_15_	950.6780	484.3562	13.1
30	TriHexCer(t18:1/20:0-OH)	C_56_H_105_NO_20_	1112.7308	565.3826	12.4
31	TetraHexCer(t18:1/20:0-OH)	C_62_H_115_NO_25_	1274.7836	646.4090	11.8
32	PentaHexCer(t18:1/20:0-OH)	C_68_H_125_NO_30_	1436.8364	727.4354	11.3
33	DiHexCer(t18:1/18:0-OH)	C_48_H_91_NO_15_	922.6467	470.3405	11.4
34	TriHexCer(t18:1/18:0-OH)	C_54_H_101_NO_20_	1084.6995	551.3669	10.6
35	TetraHexCer(t18:1/18:0-OH)	C_60_H_111_NO_25_	1246.7523	632.3933	10.1
36	DiHexCer(t18:1/16:0-OH)	C_46_H_87_NO_15_	894.6154	456.3249	9.4
37	TriHexCer(t18:1/16:0-OH)	C_52_H_97_NO_20_	1056.6682	537.3513	8.7
38	TetraHexCer(t18:1/16:0-OH)	C_58_H_107_NO_25_	1218.7210	618.3777	8.1
39	PentaHexCer(t18:1/16:0-OH)	C_64_H_117_NO_30_	1380.7738	699.4041	7.4
40	DiHexCer(d18:2/16:0-OH)	C_46_H_85_NO_14_	876.6048	447.3196	10.2
41	TriHexCer(d18:2/16:0-OH)	C_52_H_95_NO_19_	1038.6578	528.3461	9.4
42	TetraHexCer(d18:2/16:0-OH)	C_58_H_105_NO_24_	1200.7108	609.3726	8.8
43	DiHexCer(d18:2/24:1-OH)	C_54_H_99_NO_14_	986.7144	502.3744	15.3
44	TriHexCer(d18:2/24:1-OH)	C_60_H_109_NO_19_	1148.7674	583.4009	14.6
45	TetraHexCer(d18:2/24:1-OH)	C_66_H_119_NO_24_	1310.8204	664.4274	14.0

We found OHCs to have a remarkably different molecular composition compared to the sphingolipid classes, HexCer and ceramide. Previously, we characterized HexCers and confirmed the predominant LCB to be d18:2, in 23 HexCers, followed by t18:1, d18:1 and t18:0 present in 15, 5 and 4 species, respectively, matching the reported barley HexCer profiles^12^. Contrastingly, in OHCs, we found t18:1 to be the predominant LCB with d18:2 a minor LCB. The LCB t18:0, which is the most abundant LCB in barley ceramide species was not found in any of the OHCs identified^12^. Characterization of the fatty acyl chain of the ceramide backbone, found six non-hydroxyl FAs (NFAs) and 17 hydroxyl FAs (HFAs) in barley HexCers; while only a limited subset of 11 FAs were detected in OHCs and all of them were hydroxylated. Moreover, most FAs in OHCs are very long-chain fatty acids (VLCFAs). Among them, hydroxylated C24:1 has the largest intensities among all series of OHCs. Hydroxylated C16:1, which is the most abundant FA in HexCers was detected but at much lower abundance.

### Identification of barley root OHC hexosyl residue

**Figure 5:**
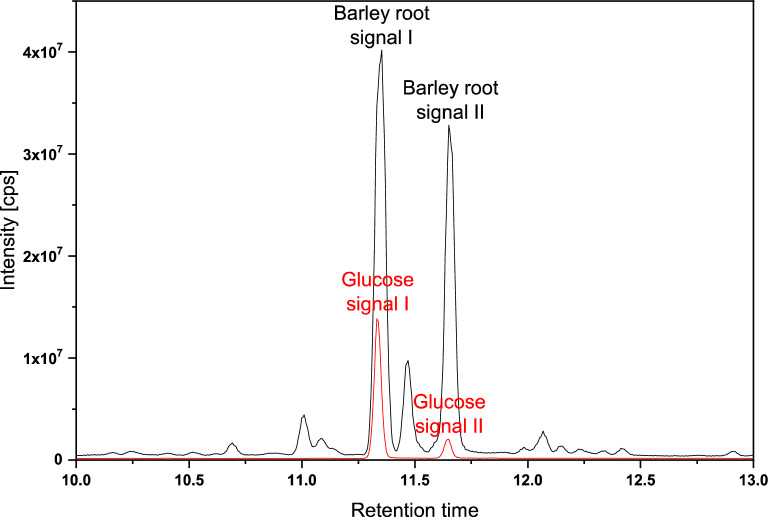
GC–MS Chromatogram of hydrolysed barley root OHC monosaccharide units after methoxymation and trimethylsilation showing matching Retention Time to glucose standards.

To identify OHC hexose composition, lipids of barley roots were separated by TLC after hydrolysis of the glycerolipids and all bands corresponding to OHCs were scraped out and extracted as a single fraction for further analysis. The headgroup of the OHCs was hydrolyzed into individual monosaccharides for further analysis via GC–MS^21^. Only one hexose type was detected, Fig. 5, confirmed as glucose by comparison with an external glucose standard. Thus, the headgroups of OHCs in barley consist of primarily glucose di- to pentamers. Other hexoses may be present in very small quantities (at least 1000 times less than glucose). However, their quantities are below the detection limit of the GC–MS analysis but could have been distinguished from glucose by both gas chromatography and mass spectrometry.

### Prevalence of OHCs in other plant species

The sphingolipidome has been comprehensively characterised in several important model plant species including *Arabidopsis* (*Arabidopsis thaliana* L.)*,* tobacco (*Nicotiana tabacum* L.), the cereals rice (*Oryza sativa* L.), bread wheat (*Triticum aestivum* L.) and moss (*Physcomitrium patens*)^1,18,22–27^. Surprisingly, all of these studies focused on Cers, GluCers and/or GIPCs, but none reported on the presence of OHCs. To understand how widely OHCs are present across the plant kingdom, we investigated different tissues of 13 diverse plant species from seven plant families. Specifically, samples were from mature seeds of barley (*Hordeum vulgare* L.), bread wheat and rice; roots and leaves of barley, wheat, rice, Italian ryegrass* (Lolium multiflorum* L.*)*, Brachypodium (*Brachypodium distachyon* L.), Arabidopsis, tobacco, chia (*Salvia hispanica* L.), chickpea (*Cicer arietinum* L.), alfalfa (*Medicago saliva* L.), lotus (*Nymphaea alba* L.) and soybean (*Glycine max* L. Merr.); as well as thallus and gemma of liverwort (*Marchantia polymorpha* L.) (Table 3).

**Table 3:** Results of survey on presence of OHCs in various tissues from 13 plant species.

Plant species	Plant family	Group	Plant tissue			
			Seed	Seedling	Root	Leaf
Barley	Poaceae	Monocot	**√**	**√**	**√**	**√**
Bread wheat	Poaceae	Monocot	**√**	**√**	**√**	**√**
Rice	Poaceae	Monocot	**√**	–	**√**	**√**
Italian ryegrass	Poaceae	Monocot	–	–	**√**	**√**
Brachypodium	Poaceae	Monocot	–	–	**√**	**√**
Arabidopsis	Brassicaceae	Dicot	–	N.D	N.D	N.D
Tobacco	Solanaceae	Dicot	–	–	–	N.D
Chickpea	Fabaceae	Dicot	–	–	N.D	N.D
Chia	Lamiaceae	Dicot	–	–	N.D	N.D
Alfalfa	Fabaceae	Dicot	–	–	N.D	N.D
Lotus	Nelumbonaceae	Dicot	–	–	N.D	N.D
Soybean	Fabaceae	Dicot	–	–	N.D	N.D

Plant species	Plant family	Group	Plant tissue			
			Thallus	Gemma	Root	Leaf
liverwort	Marchantiaceae	Non-vascular	N.D	N.D		

Their plant family, group of monocot/dicot are listed. √: confirmed presence.

*N.D.* not detected, – not tested.

Di-, Tri-, Tetra- and PentaHexCers were detected in seeds, roots and leaves of barley, bread wheat, rice*,* Italian ryegrass and Brachypodium; while no OHCs were found in any of the tissues of the other eight plant species (Table 3). Barley, wheat, rice, Italian ryegrass and Brachypodium are all monocots and belong to the grass family (Poaceae); while Arabidopsis, tobacco, chia, chickpea, alfalfa, lotus and soybean are flowering dicots from different plant families i.e., Lamiaceae, Fabaceae, Nelumbonaceae, Solanaceae and Brassicaceae. Additionally, no OHC was detected in liverwort from Marchantiaceae family (Table 3). From these results we conclude that OHCs are predominantly distributed among grasses (Poaceae).

### Changes in OHC composition of barley roots during salt stress

**Figure 6:**
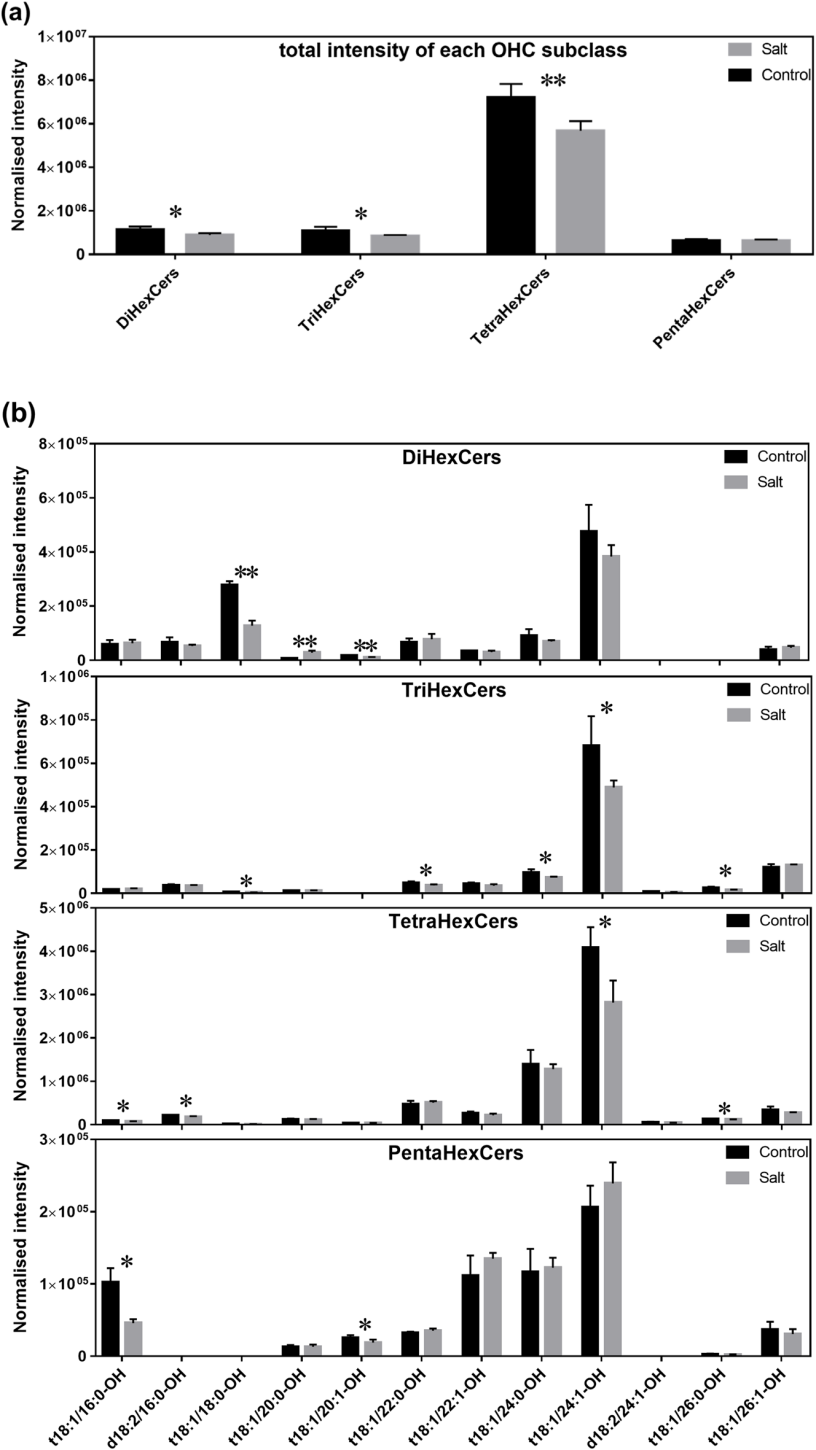
Normalised intensities of total (**a**) and individual (**b**) dihexosylceramide (DiHexCer), trihexosylceramide (TriHexCer), tetrahexosylceramide (TetraHexCer) and pentahexosylceramide (PentaHexCer) in control and salt-treated barley root extracts (*n* = 4). Black bars correspond to control group; grey bars correspond to salt-treated group. Peak area is normalised to the value equivalent to 250 mg fresh barley weight. Significance was evaluated by the Student’s *t*-test followed by Benjamini–Hochberg false discovery rate (FDR) correction; *p < 0.05; **p < 0.01; Error bars are calculate as mean + SD.

Plants vary greatly in their responses to salinity. For instance, Arabidopsis can only thrive when exposed to NaCl concentrations below 100 mM, whereas barley can endure salinity levels of up to 300 mM NaCl. Increasing evidence has suggested that the lipid composition of plant tissues may be associated with the ability of plants to tolerate salt^28–30^. In our previous studies, barley root lipid composition is altered under high salinity stress^12^; but it remains unclear if OHCs also play a role in responses to salt stress. In this study, barley root lipid extracts of control and salt-treated roots were analysed for 45 OHC lipids. 42 out of 45 OHCs were detected with CVs of intensities below 25% among five injections of a pooled biological quality control (PBQC) sample (Fig. 6, Supplementary Table S1). Total intensities of Di-, Tri-, Tetra- and PentaHexCer in control and salt-treated samples were summed and compared to show the global change of each subclass (Fig. 6a). As a result, the amount of DiHexCers, TriHexCers and TetraHexCer showed statistically significant decreases to 78%, 78% and 79% respectively in salt-treated samples; while PentaHexCer showed no change in salt-treated samples compared to control ones.

Compositional changes in 14 species including three DiHexCers, five TriHexCers, four TetraHexCers and two PentaHexCers showed significant differences (*p* < 0.05) between control and salt-treated samples (Fig. 6b). Most of the 14 OHCs experienced a decrease under salt stress with the exception of DiHexCer(t18:1/20:0-OH), which increased more than three-fold in salt-treated barley roots. TetraHexCer(t18:1/24:1-OH), which is the most abundant OHC species detected in our study, decreased 31% in salt-treated barley roots compared to control. Similarly, a strong decrease in salt-treated extract was also observed for TriHexCer(t18:1/24:1-OH) (28%, *p* < 0.05) and DiHexCer(t18:1/24:1-OH) (19%); only PentaHexCer(t18:1/24:1-OH) displayed a different tendency with a slight increase (1.16-fold). Overall, salinity caused alterations of the OHC profile in barley root with a general decrease for most OHC species.

## Discussion

OHCs discovered in the 1980’s have attracted very little research^9,10^. Recent reviews of plant sphingolipid metabolism have focused upon GlcCer and GIPCs with no mention of OHCs^2,3,6,31^. One possible reason is important model plants such as Arabidopsis and tobacco, and other dicots, do not possess this type of complex sphingolipid. In addition, a lack of modern rapid high-throughput methods to structurally characterise, profile, and semi-quantitatively measure these species have likely contributed to the lack of research. In this study, we provide evidence that OHCs are present in the most important cereal crops: wheat, rice and barley, as well as other grasses but not in any other plant family tested.

To understand more about this class of lipids, we developed a comprehensive PRM based assay enabling the identification and profiling of 45 OHCs in barley (*Hordeum vulgare* L.), including elucidation of their FA/LCB/sugar residue compositions. Isolation was accomplished by a simple monophasic extraction using relatively small amounts of fresh tissue (~ 250 mg). Analysis using modern highly sensitive mass spectrometry assays, allowed for fast preparation, detection and characterization, compared to prior methods requiring large amounts of fresh tissues (kilograms), significant preparation time and decoupled assays. We chose PRM assays, as the approach possesses the ability to perform both quantification and structural elucidation simultaneously. By combining full-scan MS1 with MS/MS data collection and targeted quantification, PRM provides detailed insights into the chemical structure of OHCs through the observation of characteristic fragment ions, ensuring accurate quantification and detailed insights into composition and arrangement of OHCs.

We found the ceramide backbones of 45 OHCs in barley contain predominately monounsaturated trihydroxy and some bis-unsaturated dihydroxy LCBs associated exclusively with hydroxylated FAs with a bias to very-long chain species; the oligohexosyl residues carry two-five glucose units in a 1 → 4 linked linear structure. TetraHexCers far outstripped the presence of Di-, Tri- and PentaHexCers combined. The highly glycosylated pentahexosyl species have not been previously reported. Whilst prior reports have indicated a more complex series of sugar residues in wheat^9^ and rice grains^10^, complementary head-group analysis by GC–MS identified the oligohexosyl headgroup in barley to be predominantly comprised of glucose. Why barley contains mainly Glu versus more complex combinations is still unknown. Other grass species will require additional headgroup analysis to identify the full range of sugar residues found in OHCs. We hypothesise, that the contrasting and distinct profile of FAs and LCBs between OHCs and HexCers indicates that the biosynthesis of OHCs is likely to be regulated by distinct mechanisms compared to HexCers. The bias to VLCFA, C22-C26 species, with a dominant C24:1-OH, high glycosylation and presence in a range of tissues may suggest a more stable sphingolipid species indicating a role in membrane structure, however, the cellular localization of these species will need to be determined in future studies to provide further insight into any specific roles. We speculated OHCs may have some role in response to abiotic stress, thus investigated compositional changes of OHCs in barley root under salt stress. An overall small decrease in OHCs was observed, which indicates that they likely play only a minor role in salt tolerance. Mechanisms underlying the impact of salinity stress on OHCs will need to be further elucidated to uncover their biological roles under salinity stress. What role/s OHCs may play in other related abiotic stress such as temperature, drought or water logging also remains an open question and will require future research^32–34^. More broadly, there is increasing evidence that lipid glycosylation plays important roles in plant interactions with microbes. Notably, sphingolipid glycosylation, specifically involving GIPC, is essential for synthesizing a specialized host-derived membrane that facilitates plant–microbe interactions, promoting endosymbiont persistence^35^. Given the high level of glycosylation, OHCs may play some role in this area but will require future research.

In conclusion, our investigation has led to the discovery of OHCs in barley via the generation and application of a new PRM assay, enabling us to rapidly identify, profile and structurally characterise the range of OHCs in barley. The outcome represents a significant step forward in our understanding of the chemical composition of this class of lipids. Expanding upon our results, we found OHCs are likely limited to monocot grasses (Poaceae), which partially explains the lack of research examining this lipid species and consequently the highly limited understanding of the biological role/s of OHCs. Biological roles remain an open question with future research needed to elucidate their specific functions. Examination in non-model species such as barley or wheat will be essential, the current availability of cereal crop genomes and the development of CRISPR technologies in cereals, when combined with our new analytical methodology will enable future elucidation of the functions of OHCs. Overall, this study adds to the ever-growing information of plant metabolic biodiversity through the discovery of a new class of lipids that from our results appears to be predominantly present in the grass family of monocots.

## Materials and methods

### Chemicals

Methanol (LC, LC–MS grade), Ammonium Acetate (purity ≥ 99%, LC–MS grade) and chloroform (Analytical grade) were purchased from Fisher Scientific (Scoresby, VIC, Australia); Hexane (LC grade) was from Honeywell (Taren Point, NSW, Australia); 2-propanol (LC–MS grade) was from RCI Labscan (Bangkok, Thailand). Deionised water was filtered through a Millipore Milli-Q system (Billerica, MA, USA). All other chemicals were purchased from Sigma-Aldrich (Castle Hill, NSW, Australia).

### Nomenclature

Lipid nomenclature used across the manuscript follows the “Comprehensive Classification System for Lipids” presented by the International Lipid Classification and Nomenclature Committee (ILCNC)^36^. The nomenclature can be viewed online on the LIPID MAPS website (http://www.lipidmaps.org). Structural information gained from mass spectrometry is insufficient to cover the precise structural information of LIPID MAPS nomenclature, requiring the use of an additional notation for simplified mass spectrometry-based information. Here we adopted the simplified notation developed by Liebisch et al. with slight modifications^37^. For example, the nomenclature HexCer(t18:1/24:1-OH) designated a hexosylceramide containing a 24-carbon dihydroxyl monounsaturated fatty acyl chain (24:1-OH), a trihydroxy monounsaturated LCB (t18:1) and hexosyl head group; while the exact positions of unsaturation and hydroxylation on either FA or LCB are unknown. The fragmentation nomenclature of glycosphingolipids was adapted and slightly modified from previous literature^38,39^.

### Plant materials

Seeds of Australian barley (*Hordeum vulgare* L.) feed variety *Mundah* were provided by the University of Adelaide (South Australia, Australia). Barley was grown in control and salt-treated conditions in hydroponics for 5 weeks as described previously with a minimum of four biological replicates per group^40^. Salt treatment was implemented with a concentration of 250 mM NaCl in hydroponics solution for the last three weeks before harvesting. Fresh tissues of *Arabidopsis* (*Arabidopsis thaliana* L.), tobacco (*Nicotiana tabacum* L.), the cereals rice (*Oryza sativa* L.), bread wheat (*Triticum aestivum* L.) and moss (*Physcomitrium patens*) were obtained from LaTrobe University (Victoria, Australia). Where relevant all permissions or licences for collection of plant materials were obtained. All methods complied with local, national and international guidelines and legislation.

When harvested, roots and mature leaves for OHC profiling were quickly separated from shoots with sterilised scissors, gently washed with distilled water to remove remaining hydroponics solution, snap frozen in liquid nitrogen and stored at − 80 °C until extraction for OHC characterisation and profiling.

### Lipid extraction

The extraction followed the procedure described previously^12,41^ with minor modifications. Frozen plant tissue was ground with a mortar and pestle into fine powder in the presence of liquid nitrogen. 250 mg of resultant powder was quickly suspended within a monophasic mixture of 2-propanol/hexane/water 60:26:14 (v/v/v, 6 mL) in KIMAX glass tube (Thomas Scientific) and incubated at 60 °C for 30 min in an Eppendorf Thermomixer Comfort (Hamburg, Germany) at 500 rpm. Samples were vortexed for 10 s and sonicated for 1 min every 10 min during incubation. The extract was centrifuged at 1400* g* for 20 min at room temperature. The supernatant was transferred to a new tube, evaporated to dryness under a stream of nitrogen, then re-constituted in 500 μL of 2-propanol/methanol/water 4:4:1 (v/v/v) and stored at − 20 °C. Glass pipettes were used for transfer of organic solvents except for the reconstitution. A pooled biological quality control (PBQC) sample produced by collecting 100 μL of each extract was used to monitor the stability of the analysis. Two blank sample (30% Methanol) injections and one PBQC sample injection were carried at the start, end and every four to five samples throughout the data acquisition.

### OHC characterisation and profiling using HPLC–ESI–QqTOF

Chromatographic separation of OHCs was performed on an Agilent 1290 series system (Santa Clara, CA, USA) using an Agilent ZORBAX Eclipse XDB C18 (100 mm × 2.1 mm, 1.8 µm) column at a flow rate of 0.20 mL/min at 50 °C. A programmed gradient elution based on two mobile phases was applied as follows: mobile phase A, methanol/20 mM ammonium acetate 3:7 (v/v); and mobile phase B, 2-propanol/methanol/20 mM ammonium acetate 6:3:1 (v/v/v). The gradient started with 40% B for 2 min, increased from 40 to 100% B for 8 min and held at 100% B for 14 min, and decreased from 100 to 40% for 0.5 min and held at 40% for 2 min.

Detection of OHCs was carried out on a SCIEX TripleTOF™ 6600 quadrupole time-of-flight (QqTOF) mass analyser (Framingham, MA, USA) using parallel reaction monitoring (PRM) assays. PRM assays were composed of a MS1 experiment (250 ms accumulation time, scan range: 100–1800 Da) followed by different numbers of targeted MS/MS experiments (50 ms accumulation time, scan range: 100–1800 Da). All MS1 experiments were performed in high-resolution mode (~ 35,000 FWHM) and all MS/MS experiments were performed in high-sensitivity mode (~ 20,000 FWHM). Parameters of targeted precursor information on PRM assays including *m/z*, predicted RTs and RT window width were entered to the 6600 TripleTOF™ Analyst acquisition software (Version 2.2) using Skyline software as described previously^42^.

Other general ESI parameters were optimized and pre-set for all measurements as follows: Source temperature, 450 °C; Curtain gas, 30 psi; Gas 1, 45 psi; Gas 2, 45 psi; Declustering potential (DP): + 100 V in positive ion mode and − 150 V in negative ion mode; Ion spray voltage floating (ISVF) was set to − 4500 V in negative ion mode and + 5500 V in positive ion mode. Instrument was calibrated automatically via the calibrant delivery system (CDS) which automatically released atmospheric pressure chemical ionization (APCI) calibration solution (Foster City, CA, USA) every 10 samples. CDS injected either positive or negative APCI calibration solution depending on the polarity of the ESI and calibrated the mass accuracy of the 6600 TripleTOF™ system in both ionisation modes including TOF–MS and high-sensitivity MS/MS. Actual mass accuracy was below 5 ppm in MS1 spectra and 15 ppm in MS/MS spectra.

### Data visualisation and processing

PeakView software (Version 1.2, SCIEX, Framingham, Massachusetts, USA) was used to visualise MS1 and MS/MS spectra. Lipid quantification using MS/MS data in PRM assays was based on the peak area of extracted ion chromatogram for precursor ions in MultiQuant (Version 3.0.2). Peak picking for precursor ions was set to 50 ppm width. Integration settings were as follows: Noise percentage = 40%; Gaussian smooth width = 2 points.

Intensities of OHCs in each sample (control and salt-treated) were acquired and normalised to 250 mg sample fresh weight. Student’s *t*-tests were conducted to evaluate for significance (*p*-value) of differences in mean concentration between treatment groups (n = 4/group) using GraphPad Prism (Version 7.0). Adjusted *p*-values were obtained with Benjamini–Hochberg false discovery rate (FDR) correction.

Supplementary Fig. S1 created with BioRender.com.

### Characterization of the head group identity of OHCs

Total lipids were extracted from 20 mg freeze-dried barley roots with 2-propanol/hexane/water 60:26:14 (v/v/v) using previously published methods^43^ and dissolved in 0.8 ml of tetrahydrofuran/methanol/water (TMW) 4:4:1 (v/v/v). The sample was then treated with methylamine^44^ to hydrolyse the glycerolipids and afterwards redissolved in 800 µl TMW. To purify the OHCs by thin layer chromatography (TLC), the remaining lipids were separated on a silica gel 60 TLC plate (Merck, Darmstadt, Germany) with chloroform/methanol/water 60:35:8 (v/v/v) and visualized under UV after spraying with 0.2% (w/v) anilinonaphthalene-sulphonate. The silica material containing those lipid bands which migrated with and slightly below the commercial lipid standard *D*-glucosyl-ß-1,1′-*N*-lauroyl-*D*-*erythro*-sphingosine (Avanti Polar Lipids, Alabaster, AL, USA) was scraped from the TLC plate (corresponding to the bands of Mono-, Di-, Tri- and PentaHexCers), lipids were extracted in 1 ml of chloroform/methanol/water 60:35:8 (v/v/v) and evaporated under a stream of nitrogen. Monosaccharide hydrolysis and analysis was adapted from Guzha et al.^21^. After hydrolysis with trifluoracetic acid, hydrolysis products were dissolved in water then evaporated to dryness to remove residual acid. The following monosaccharide analysis was performed by gas chromatography–mass spectrometry after derivatization with methoxyamide and *N*-methyl-*N*-(trimethylsilyl)trifluoroacetamide using an Agilent 7890B GC system coupled to an Agilent 5977B mass selective detector equipped with a capillary HP-5MS UI column (30 m × 0.25 mm; 0.25 µm coating thickness; Agilent) operated with MSD ChemStation Data Analysis (F.01.03.2357). Data was analysed using Agilent MassHunter software (B.07.05).

### Supplementary Information

Supplementary Figure S1.

Supplementary Table S1.

Supplementary Table S2.

## Data Availability

All datasets generated are contained within the manuscript or supplementary materials. Additional, raw mass spectrometry data files used for identification of OHCs and species profiling are available upon request from DY (dyu@svi.edu.au).
