# Supplementary material for: Discovery of novel neutral glycosphingolipids in cereal crops: rapid profiling using reversed-phased HPLC–ESI–QqTOF with parallel reaction monitoring
Source: Sci Rep. 2023 Dec 19;13:22560. doi: 10.1038/s41598-023-49981-7 (PMC10728066; doi:10.1038/s41598-023-49981-7)
Supplement: Supplementary file 1 — Supplementary Figure S1. [file 41598_2023_49981_MOESM1_ESM.pdf]

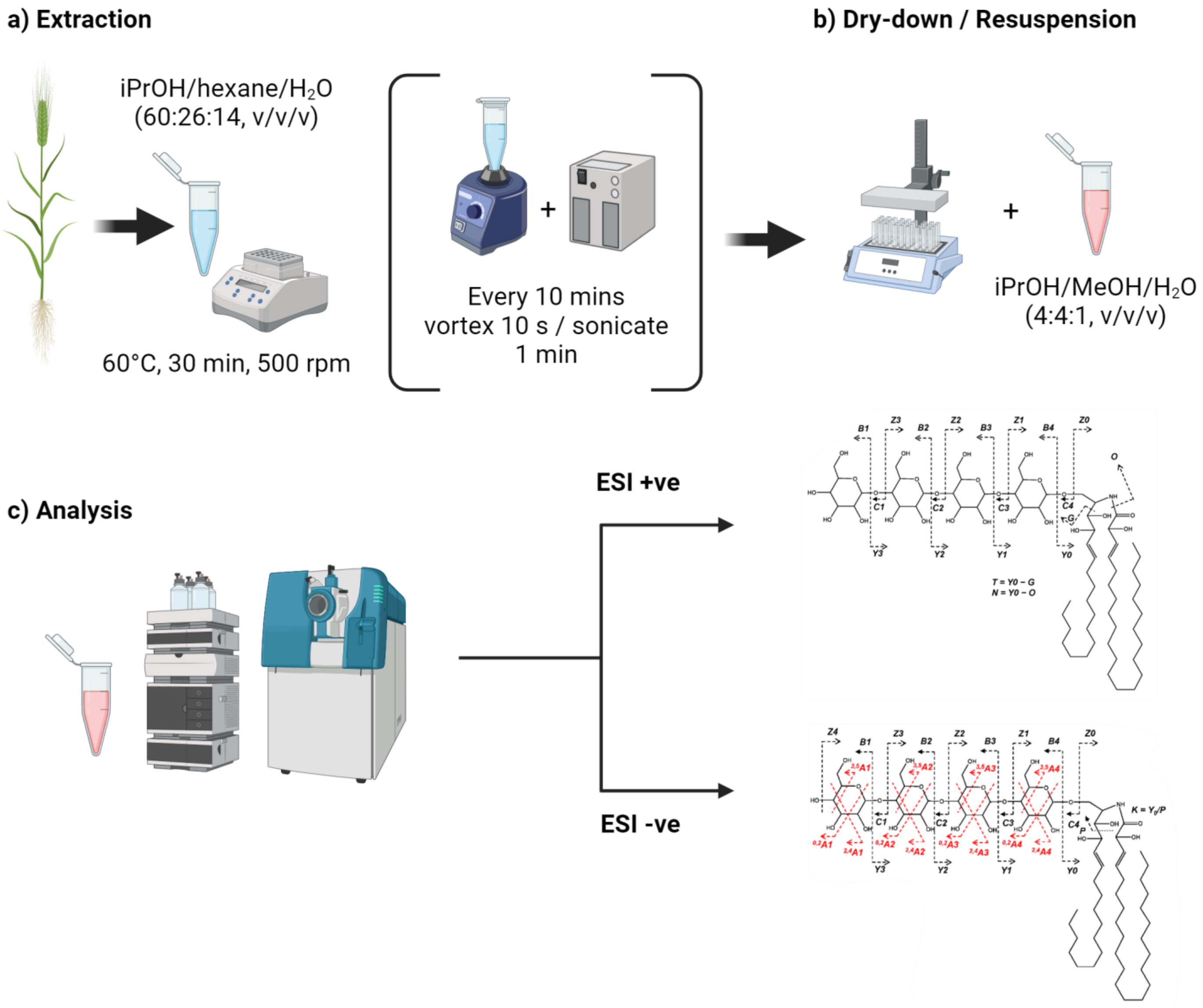

Supplementary Figure 1: Workflow depicting sample preparation and analysis by RP-HPLC-QqTOF-MS/MS. 1) Extraction by suspending frozen tissues in iPrOH/hexane/H_2O (60:26:12 v/v/v) followed by heating and mixing at 60°C (30 min, 500 rpm). Every 10 min, the samples were vortexed (10 s) and sonicated (1 min) prior to 2) nitrogen blow-down and resuspension in iPrOH/MeOH/H_2O (4:4:1, v/v/v). 3) Samples analysis by LC-ESI-QqTOF in both +ve providing an analysis of the parent species and -ve ion modes providing detailed linkage analysis. Created with BioRender.com
